# A panel of 8-lncRNA predicts prognosis of breast cancer patients and migration of breast cancer cells

**DOI:** 10.1371/journal.pone.0249174

**Published:** 2021-06-04

**Authors:** Lili Zhu, Kang Cui, Lanling Weng, Pu Yu, Yabing Du, Tengfei Zhang, Hong Liu, Bingjie Li, Wang Ma

**Affiliations:** 1 Department of Oncology, the First Affiliated Hospital of Zhengzhou University, Zhengzhou, China; 2 Academy of Medical Science, Zhengzhou University, Zhengzhou, China; Universitat de Barcelona, SPAIN

## Abstract

**Background:**

Breast cancer (BCa) is the most commonly diagnosed cancer and the leading cause of cancer death among females around the world. Recent studies have indicated that long non-coding RNAs (lncRNAs) can serve as an independent biomarker for diagnosis and prognosis in many types of cancer, including pancreatic adenocarcinoma, gastric cancer, liver cancer, and lung cancer. Previous studies have shown that many lncRNAs are associated with the occurrence and development of BCa. However, few studies have combined multiple lncRNAs to predict the prognosis of early-stage BCa patients.

**Methods:**

Systematic and comprehensive analysis of data from The Cancer Genome Atlas (TCGA) was conducted to identify lncRNA signatures with prognostic value in BCa. Additionally, the relative expression levels of the 8 lncRNA of several BCa cell lines were detected by quantitative real-time PCR (qPCR) and the results were substituted into a risk score formula. Finally, migration assays were used to verify the result from prognostic analysis according to the risk scores among cell lines with different risk scores.

**Results:**

Our study included 808 BCa patients with complete clinical data. A panel of 8 lncRNAs was identified using Wilcox tests as different between normal and tumor tissue of the BCa patients. This panel was used to analyze the survival of BCa patients. Patients with low risk scores had greater overall survival (OS) than those with high risk scores. Multivariate Cox regression analyses demonstrated that the lncRNA signature was an independent prognostic factor. Gene Set Enrichment Analysis (GSEA) suggested that the lncRNAs might be involved in several molecular signaling pathways implicated in BCa such as the DNA replication pathway, the cell cycle pathway, and the pentose phosphate pathway. Validation experiments in breast cancer cells to test cell migration by using wound-healing assays supported the results of the model.

**Conclusion:**

Our study demonstrated that a panel of 8 lncRNAs has the potential to be used as an independent prognostic biomarker of BCa.

## Introduction

Breast cancer is the most commonly diagnosed tumor and the leading cause of cancer death among females around the world, accounting for 24% of all diagnosed cancers and 15% of all cancer death in females [1]. According to Global Cancer Statistics, in the year 2018 there were nearly 2.1 million new cases diagnosed globally and approximately 626,679 cancer deaths [2]. In recent years, the incidence of breast cancer has been increasing annually in most developing countries, with half of all new breast cancer diagnoses and nearly 60% of breast cancer deaths occurring in developing countries. The morbidity and mortality of BCa represents a major global health burden. In recent years, the optimization of treatments, including endocrine therapy, chemotherapy, radiotherapy, targeted therapy, immunotherapy, and Traditional Chinese Medicine, has improved the prognosis of breast cancer patients [3–6]. However, a significant problem remains local recurrence and distant metastasis after months or years, even in patients with early diagnosis and treatment [7].

Previous studies have divided patients into two groups to predict prognosis of BCa based on the traditional pathological characteristics of the tumor, including tumor status, lymph node status and grade, and positive resection margins [8]. However, inconsistencies often exist between these indicators and survival. For example, due to molecular differences, clinical outcomes can be very different even in patients with histologically similar tumors [9]. Therefore, the evaluation of prognostic indicators of BCa patients has gradually shifted to molecular research. However, the biomarkers that have been found using these methods may not be currently sufficient to accurately predict BCa prognosis. Cancer is a complex disease, due to the accumulation of multiple genetic mutations, which triggers somatic cell carcinogenesis. Therefore, the detection of differential gene expression between normal tissues and tumor tissues has clinical significance. The era of big data has coincided with significant technological advances. It is now possible to use existing database resources to conduct integrated analysis of differential gene expression and find a panel of signatures associated with the prognosis of patients with early cancer [10].

Analysis of the human transcriptome has indicated that more than 50% of transcripts have no protein-coding potential [11, 12]. Long non-coding RNAs (lncRNAs) are transcripts with a length of more than 200 nucleotides [13, 14]. Increasing evidence indicates that lncRNAs play important roles in regulating gene expression at the transcriptional, posttranscriptional, and chromosomal levels and are associated with a large range of biological processes including transcriptional regulation, cell growth, and tumorigenesis [15–18]. In the past few years, lncRNAs have served as biomarkers for diagnosis and prognosis in a variety of cancers, including nasopharyngeal carcinoma, gastric cancer, non-small cell lung cancer, diffuse large B cell lymphoma, BCa, and ovarian cancer [19–26]. In BCa, several lncRNAs have been associated with prognosis, including RUSC1-AS-N, LINP1, MIAT, CAT104, LINC01234, STXBP5-AS1, and MALAT1 [27–31]. However, most of these studies only focused on a small number of genes, and few studies have combined multiple lncRNAs to predict survival rates of BCa patients [32, 33].

In this study, we analyzed the transcriptome results of normal tissues and tumor tissues of BCa patients obtained from the TCGA database (TCGA, http://cancergenome.nih.gov/) to find differences in expressed lncRNA genes, and then we constructed a multi-lncRNA-based signature and developed a formula to predict the prognosis of BCa. Finally, we conducted a verification of the signature using cytological migration assays. Our results indicate that this panel of lncRNA signatures could serve as an effective, independent prognostic biomarker for patients with BCa.

## Material and methods

### Expression profiles and sample information

In order to find differences in gene expression, the RNA-seq of tumor tissue and normal tissue files and clinical features of BCa patients were retrieved from TCGA data and analysed. In order to obtain lncRNAs specific for BCa, patients with other diseases were excluded and only patients with a single BCa disease were retained. Thus, a total of 808 BCa patients with clinical data and without other malignancies were involved in our study. The corresponding clinical information including age, tumor-status, surgical status, and TNM grade were recorded. The lncRNAs derived from TCGA data were annotated using Ensemble ID from the GENCODE project [34]. The expressed lncRNAs were defined as those with an average reads per kilobase per million mapped reads (RPKM) ≥ 0.3 across all 808 BCa patients. As the data was downloaded from TCGA, further approval by an ethics committee was not needed. Data processing was performed in accordance with the TCGA human subject protection and data access policies.

### Differentially expressed gene analysis

We used Wilcox tests to determine the differentially expressed genes (DEGs) between normal and tumor tissues (|logFC|>2 & fdr<0.05). The relationship between the expression level of each lncRNA and the OS of BCa patients was evaluated by Cox regression analysis using the “survminer” R package for genes chosen from multiple factors cox regression analysis [35]. The genes with hazard ratio (HR) values less than 1 represent protective genes, and the genes with HR values greater than 1 represent increased-risk genes. A multi-factor Cox model was constructed using the “survival” R package for genes selected for single factor significance, and the optimal model was found based on the Akaike Information Criterion (AIC): risk score = β1* gene1 +β2*gene2 + …βx*genex. Using the median risk score as the cutoff point, BCa patients were divided into a high score and a low score group.

The prognostic value of clinical variables and the lncRNA risk score on OS of BCa patients was initially assessed using univariate Cox proportional hazards regression analyses. Subsequently, each variable identified via univariate analysis was evaluated by multivariate Cox proportional hazards regression analysis. The survival curves of the high and low risk groups were compared using the Kaplan-Meier method, and the statistical method was log-rank. The association of the lncRNA signature and clinical variables was evaluated using Chi-square tests. The differential genes were clustered using the “pheatmap” R package [36]. The ROC curve was plotted against the difference using the “survival” R package and the AUC value was calculated to judge the reliability of the prediction results. All the statistical analyses were conducted with the R Project for Statistical Computing, BRB-Array Tools and SPSS 16.0 software (SPSS Inc., Chicago, IL, USA), as appropriate. A two-sided P value < 0.05 was defined as statistically significant unless otherwise indicated.

### Functional enrichment analysis

Pearson correlation coefficients were calculated to assess co-expressed relationships between the risky/protective lncRNAs and protein-coding genes (PCGs). The genes with corrationel coefficients higher than 0.3 and P<0.05 were identified as being co-expressed with PCGs. To evaluate the potential biological processes and pathways the lncRNAs might be involved in, functional enrichment analysis was performed based on the co-expressed PCGs for Gene Ontology (GO) biological process (BP) and Kyoto Encyclopedia of Genes and Genomes (KEGG) pathway using the DAVID Bioinformatics Tool (https://david.ncifcrf.gov/, version 6.8) [34]. The GO terms and KEGG pathways with P value< 0.01 were considered as significantly enriched function annotations.

Gene set enrichment analysis (GSEA) was carried out to explore the potential altered pathways between the high risk score and low score groups using java software GSEA (http://software.broadinstitute.org/gsea/index.jsp) [34]. A false discovery rate (FDR) value < 0.05 after 1000 random permutations was set as the cutoff criterion.

### The validation using cell lines

#### Cell lines and cell culture

Human BCa cell lines (MCF7, MDA-MB-468 and MDA-MB-231) were purchased from the Jikai Gene Company (Xu Hui, Shanghai, China). The human BCa cell line of BT20 was purchased from the Jiniou Bioscience Company (Luogang, Guangzhou, China). The normal breast cell line of MCF10A was purchased from the Meiyan Bioscience Company (Minhang, Shanghai, China). The cells of MCF7, MDA-MB-468 and BT20 were cultured using RPMI 1640 medium (Corning, America) and cells of MDA-MB-231 were cultured using L15 medium (HyClone, America). All cells were supplemented with 10% fetal bovine serum (FBS; Corning, America) and 1% streptomycin–penicillin antibiotic solution (Corning, America) at 37°C with 5% CO_2_. Cells of MCF10A were cultured using a mixed medium (Enzyme research, Shanghai, China) (DMEM/F12(1:1) medium + Horse serum(5%)+ insulin(10ug/ml)+ Epidermal growth factor (20ng/ml) + Cholera toxin (100ng/ml) + Hydrocortisone(0.5ug/ml)) at 37°C with 5% CO_2_.

#### Quantitative real-time PCR

RNA was isolated using a kit from TIANGEN (Haidian, Beijing, China), according to the manufacturer’s protocols. All primers were synthesized by Shang Ya Bio (Zhengzhou, Henan, China) and the sequences are listed in S1 Table. The qRT-PCR assays were all performed using a Quant Studio 5 Flex real-time PCR instrument (Thermo Fisher Scientific, Pudong, and Shanghai, China). GAPDH was used as an internal reference for lncRNA. The Two Step qRT-PCR Kit (Takaro, Japan) was applied to detect lncRNA and the relative expression was analyzed using the 2 ^-ΔΔCt^ method. All experiments were performed in triplicate.

#### Wound-healing and Transwell assays

Cell migratory capability was evaluated using a wound-healing assay and a transwell assay. For the wound-healing assay, the cell suspensions (2ml, 5×10^5^ cells) were seeded into 6-well plates (Corning, American), followed by overnight incubation. Next, the cell layer was scratched with a plastic tip, and the cells were subsequently cultured in serum-free medium. Photographs of wounded areas were taken using an inverted microscope (Leica MZ8, Leica Microsystems, and Wetzlar, Germany) at 0h and 24h. Each assay was performed independently in triplicate.

For the transwell assay, MCF7, MDA-MB-231, MDA-MB-468, or BT20 cells were resuspended in serum-free medium (3×10 5 cells/ml) and the suspensions (200μl) were subsequently seeded into the upper side of a transwell chamber (8μm pore size; Costar, Cambridge, MA). Then, the RPMI 1640 medium containing 10% FBS was added into the lower chambers. After 28h of incubation, the non-migratory cells on the upper side of the transwell chambers were gently removed with a cotton swab. Next, were utilized to the cells on the lower side of the chambers were fixed and stained using 4% paraformaldehyde and 0.1% crystal violet, respectively. Finally, after three washes with PBS and air-drying, photographs were taken using an inverted microscope (Leica MZ8, Leica Microsystems, Wetzlar, Germany). Five random high-powered fields of view were randomly selected in each chamber. All experiments were performed in triplicate.

#### Statistical analysis

GraphPad Prism version 8 was used for data analysis. All results were expressed as mean ±standard deviation (SD). The statistical significance was analyzed using one-way ANOVA or Student’s t-test as appropriately. P< 0.05 was considered significant.

## Results

### Data download and characteristics of patients

We downloaded level 3 RNA-seq data from the TCGA database (https://cancergenome.nih.gov/) and excluded data which did not contain complete survival time information. The remaining data was used as the research subjects for this study, and an analysis of the clinical data for these subjects is presented in Table 1. A total of 808 patients with BCa were included in this study. Among the 808 patients, 748 (92.6%) patients were tumor free, 243 (30.1%) were older than 65 years old, 573 (70.9%) were post-menopause, 250 (30.9%) and 157 (19.4%) patients had received a modified radical mastectomy or simple mastectomy, respectively. Approximately half of the patients were at Stage II in the clinical pathological staging. Each sample of the transcriptome data downloaded from TCGA had a profile, which were all combined into a matrix file. We converted the matrix using Ensemble ID into a gene symbol matrix using the ensemble database and determined the tumor tissues and normal tissues from the symbol matrix.

**Table 1:** Characteristics of the 808 breast cancer (BCa) patients in the present study.

Characteristics	Number
**All**	808
**menopause**	
Indeterminate	19(2.35%)
Peri	37(4.58%)
Post	573(70.92%)
Pre	179(22.15%)
**Tumor_status**	
Tumor free	748(92.57%)
With tumor	60(7.43%)
**Age (year)**	
< = 65	565(69.93%)
>65	243(30.07%)
**Surgical**	
Lumpectomy	207(25.62%)
Modified Radical Mastectomy	250(30.94%)
Other	194(24.01%)
Simple Mastectomy	157(19.43%)
**T**	
T1	211(26.11%)
T2	474(58.66%)
T3	97(12%)
T4	26(3.22%)
**N**	
N0	399(49.38%)
N1	260(32.18%)
N2	88(10.89%)
N3	53(6.56%)
Nx	8(0.99%)
**M**	
M0	689(85.27%)
M1	14(1.73%)
Mx	105(13%)
**Stage**	
Stage I	143(17.7%)
Stage II	470(58.17%)
Stage III	181(22.4%)
Stage IV	14(1.73%)

### Differential expression of genes (DEGs) analysis and identification of lncRNAs correlated with OS

The up-regulated DEGs, which are the genes with a higher level of expression in tumor tissues compared to normal tissues, are listed in S1 File. The down-regulated DEGs, which are the genes with a lower level of expression in tumor tissues compared to normal tissues, are listed in S2 File. The most obviously differentially expressed genes that we screened were MNX1-AS1, SIRLNT, AC092920.1, AC105219.1, AL355312.3, AC055854.1, LINC01117, and ACTA2-AS1.

**Fig 1:**
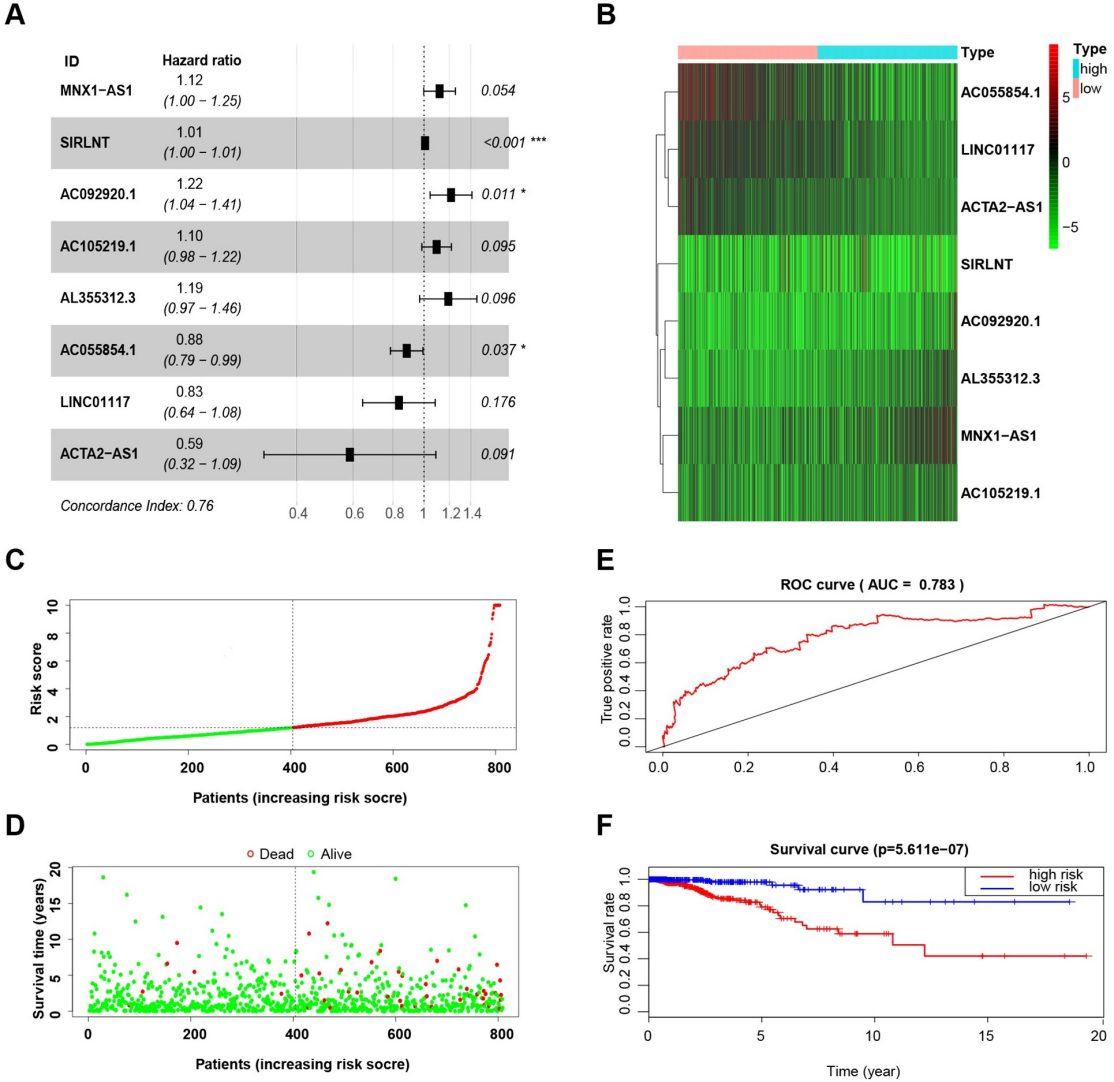
The construction of the 8-lncRNA signature and its performance analysis. (A) Forest map showing correlation between the expression levels of the 8 lncRNAs and OS in BCa patients. (B) Heatmap showing the expression levels of 8 lncRNAs in BCa patients. (C) Risk scores calculated by the expression levels of the 8 lncRNAs in BCa patients. (D) Patient survival status and follow-up time. (E) ROC curve analyzing the performance of the model using the risk scores. (F) Survival (Kaplan-Meier curve) analysis showing different outcomes between the BCa patients with low-risk and high-risk scores.

We analyzed the relationship between the lncRNAs and OS, and a forest map was created (Fig 1A). The forest map was drawn for genes chosen from multiple factors Cox regression analysis using the “survival” R package. In the forest map, the genes with HR values less than 1 represent low-risk genes, and the genes with HR values greater than 1 represent high-risk genes. Risk of patients increases as the amount of expression of high-risk genes increases and as the number of low-risk genes increases in expression, the patients’ risk decreases. As can be seen from the figure, MNX1-AS1, SIRLNT, AC092920.1, AC105219.1, and AL355312.3 are high-risk genes, while AC055854.1, LINC01117, and ACTA2-AS1 are low-risk genes. The results also showed that only three independent lncRNAs (SIRLNT, AC092920.1, and AC055854.1) were significantly correlated with OS. Therefore, individual lncRNAs do not play a more precise role in predicting OS of BCa patients than multiple lncRNAs.

### Multi-factor Cox Model construction and a panel of 8 lncRNA as an independent factor to predict BCa prognosis

A multi-factor Cox model was constructed for genes selected for single factor significance using the “survival” R package, and the optimal model was selected based on Akaike Information Criterion (AIC) value. The final selected risk model was: risk score = MNX1-AS1* (0.112) + SIRLNT* (0.008) + AC092920.1* (0.195) + AC105219.1*(0.091) + AL355312.3*(0.175) + AC055854.1*(-0.124) + LINC01117*(-0.181) + ACTA2-AS1*(-0.534).

The survival risk score of each sample was obtained according to the formula and the level of lncRNA expression of each sample (S3 File), and we then obtained the median risk score value for all samples. Using the median value as a threshold, the total sample of BCa patients were divided evenly into high risk (n = 404) and low risk (n = 404) groups. Differential genes expression was clustered using the “pheatmap” R package and is presented in Fig 1B. In the heatmap, red represents a high expression of the genes in the sample, and green represents a low expression of the genes. As shown in Fig 1C, a higher expression was observed for the protective lncRNAs in the low score group, while higher expression was noted for the increased-risk lncRNAs in the high score group. There were more deaths and a shorter survival time in the high-risk score group, as shown in Fig 1D.

The survival of the high- and low-risk groups was compared using the Kaplan-Meier method, using log-rank statistics. The survival curve based on risk group is shown in Fig 1F. The results indicate that there was a significant difference between the high- and low-risk groups (p < 0.001). The five-year survival rate was 79.1% (95% CI: 71.5%-87.6%) in the high-risk group and 97.9% (95% CI: 95.6%-100%) in the low-risk group. However, KM survival analysis based on the expression of single lncRNA indicates that any single lncRNA does not similar prognostic value as the model (S1 Fig).

### ROC curve

The ROC curve was plotted using the “survival” R package (Fig 1E) and the AUC value was calculated. Obtaining an AUC value greater than 0.7 indicates that the model is a good predictor of patient survival and the closer the AUC is to 1, the better the diagnostic effect. AUC has a lower accuracy when its value is between 0.5 and 0.7; AUC has a good accuracy if its value is between 0.7 and 0.9; and AUC has a great accuracy if its value is above 0.9. The AUC of the lncRNA model was greater than 0.7, indicating that our model was able to accurately predict the survival of BCa patients. However, the maximum AUC value of single lncRNA is 0.606, indicating the model has greater performance compared to any single lncRNA from the model (S2 Fig).

### The correlation analysis between lncRNAs and clinical traits and between clinical traits or risk score and OS of BCa patients

Analyzing the relationship between the risk score and clinical features demonstrated that a higher risk score was associated with premenopausal status, later stage, and tumor status (Table 2).

**Table 2:** Correlations of the 8 lncRNAs and the lncRNA signature risk score with clinical features in BC patients (presented as P value).

ID	Menopause (Indeterminate VS Peri VS Post VS Pre)	Tumor Status (Tumor free VS With tumor)	Age (<65 VS. ≥65)	Surgical(Lumpectomy VS Modified Radical Mastectomy VS Other VS Simple Mastectomy)	T (T1 vs T2 VS T3 VS T4)	N (N0 vs N1 VS N2 VS N3 VS Nx)	M (M0 VS M1 VS Mx)	Stage (I VS. II VS. III VS IV)
MNX1-AS1	0.001	1.000	0.125	0.955	0.001	0.131	0.765	0.008
SIRLNT	0.357	0.687	0.539	0.003	0.376	0.280	0.473	0.155
AC092920.1	0.941	0.348	0.046	0.272	0.020	0.433	0.355	0.040
AC105219.1	0.467	0.081	0.759	0.313	0.405	0.149	0.004	0.032
AL355312.3	0.158	0.081	0.539	0.164	0.982	0.447	0.743	0.905
AC055854.1	0.204	0.023	0.220	0.029	0.178	0.156	0.154	0.080
LINC01117	0.988	0.893	0.759	0.033	0.103	0.373	0.816	0.868
ACTA2-AS1	0.084	0.502	<0.001	0.002	0.002	0.211	0.012	0.031
riskScore	<0.001	0.023	0.066	0.045	0.001	0.110	0.124	0.052

Table 2 presents the results of the chi-square tests that examined the clinical correlation between each lncRNA and clinical traits. In the table, the column name indicates the lncRNA, the row name indicates the clinical trait, and the corresponding value of the association between lncRNA and clinical trait is indicated by the p value. p<0.05 indicates that the lncRNA was associated with clinical trait. MNX1-AS1 was upregulated in premenopausal patients compared with postmenopausal patients. The expression of ACTA2-AS1 was significantly correlated with age. SIRLNT and ACTA2-AS1 were associated with surgical methods, and the expression of MNX1-AS1 was associated with clinical stage.

Multivariate Cox analysis was performed to examine the relationship between OS of BCa patients and clinical variables or risk score (Table 3). The value of the risk score was significant for both the single factor and multivariate analysis, indicating that the risk score obtained by our formula was an independent predictor of survival in BCa patients (P<0.001).

**Table 3:** Cox proportional regression analysis of the correlation of clinical factors and the lncRNA signature risk score with OS in BCa patients.

Variables	Univariate analysis	P value	Multivariate analysis	P value
	HR (95%CI)		HR (95%CI)	
Menopause (Indeterminate VS Peri VS Post VS Pre)	1.036 (0.749,1.431)	0.832	0.668 (0.438,1.018)	0.061
Tumor Status (Tumor free VS With tumor)	11.869(6.384,22.065)	<0.001	13.974(6.753,28.915)	<0.001
Age (<65 VS. ≥65)	1.032 (1.009,1.057)	0.007	1.065 (1.035,1.095)	<0.001
Surgical (Lumpectomy VS Modified Radical Mastectomy VS Other VS Simple Mastectomy)	1.091 (0.829,1.437)	0.533	1.150 (0.852,1.553)	0.362
T (T1 vs T2 VS T3 VS T4)	1.365 (0.950,1.961)	0.092	0.814 (0.492,1.347)	0.423
N (N0 vs N1 VS N2 VS N3 VS Nx)	2.053 (1.640,2.737)	<0.001	1.390 (0.846,2.283)	0.194
M (M0 VS M1 VS Mx)	3.504 (1.365,8.992)	0.009	0.305 (0.074,1.249)	0.099
Stage (I VS. II VS. III VS IV)	2.297 (1.586,3.327)	<0.001	1.223 (0.518,2.889)	0.646
RiskScore (high VS. low)	1.069 (1.041,1.098)	<0.001	1.077 (1.047,1.109)	<0.001

HR: hazard ratio; CI: confidence interval.

### Co-expression analysis and GO enrichment analysis

The protein-coding genes (PCGs) were tested for correlation in order to find genes with a co-expression relationship with the lncRNA in the model. The screening conditions were (|cor|>0.3 & P<0.05). The genes with a co-expression with the lncRNAs in the model are listed in S4 File.

Gene Ontology (GO) enrichment analysis, which is often used to provide background information on gene function classification labeling and gene function research, can be divided into a number of parts, three of which are commonly used: molecular function (MF), biological process (BP), and cellular composition (CC). The gene ontology database is used to obtain GO annotation information (functional information) of the gene by searching through species and genetic information.

We used the R package “ClusterProfiler” to conduct GO analysis [37]. According to the GO annotation of each gene, we selected all the genes of the species as background genes and used statistical methods to calculate the P value. By setting the significance threshold to obtain the high frequency annotations with statistical significance relative to the background, we obtained the gene collection in the GO category, as well as distribution and significance information.

**Fig 2:**
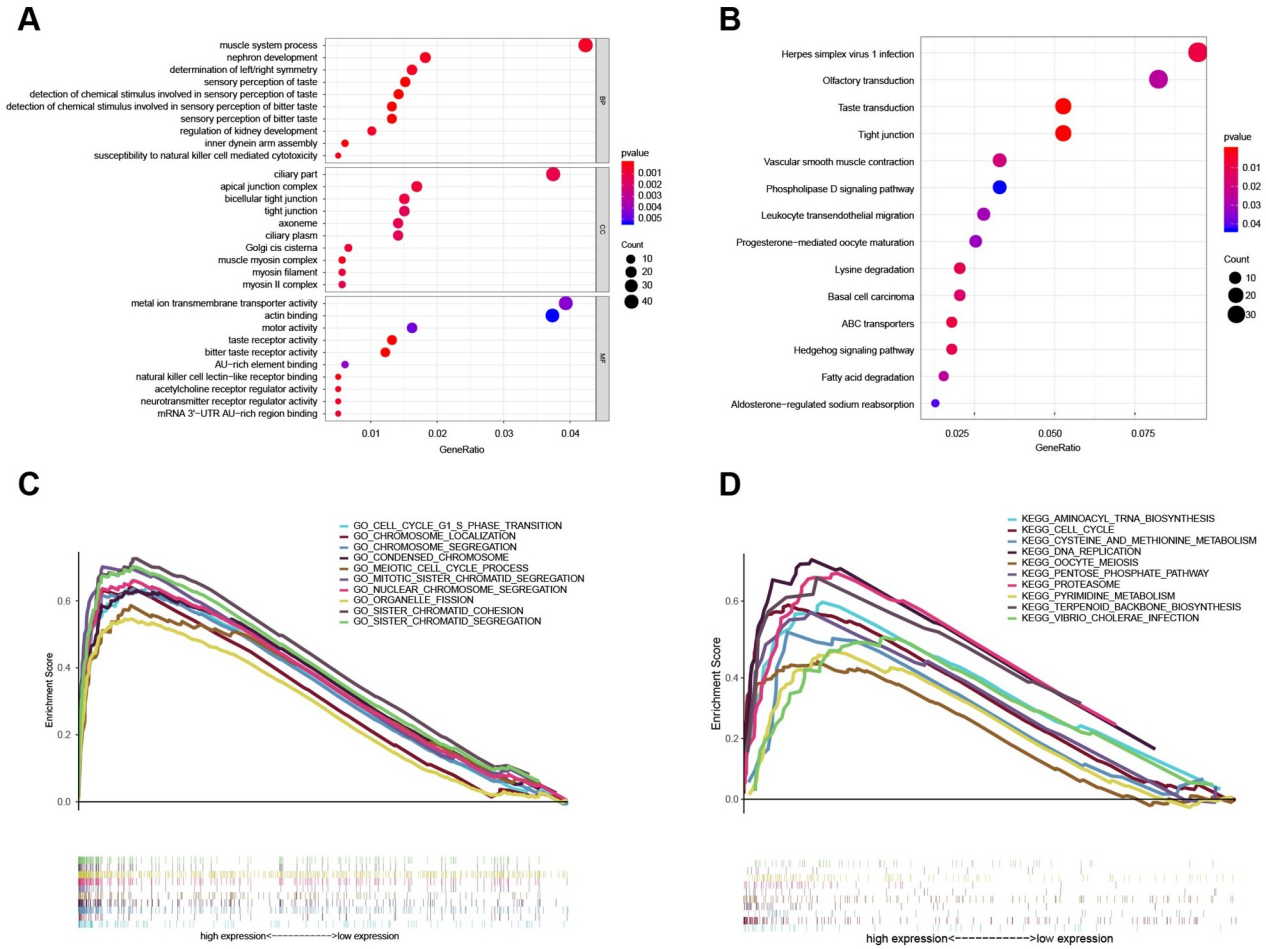
The functional enrichment analysis of the 8-lncRNA signature. (A) GO enrichment analysis, including MF, BP, and CC. (B) The result of KEGG enrichment analysis. (C), (D) GSEA-GO/KEGG enrichment analysis. GO: gene ontology; MF: molecular function; CC: cellular composition; BP: biological process; KEGG: Kyoto Encyclopedia of Genes and Genomes. GSEA: gene set enrichment analysis; BCa: breast cancer.

GO functional enrichment analysis was conducted with the co-expressed genes using cluster profiler in R, and P < 0.05 was used as a screening condition. The enrichment results are plotted as a bubble chart and shown in Fig 2A. We observed a significantly enriched GO, and a table of enrichment is presented in S5 File.

### KEGG enrichment analysis

We analyzed the results taking into consideration the complex regulatory pathways. Based on the Kyoto encyclopedia of genes and genomes (KEGG) biological pathway database (http://www.genome.jp/), a KEGG database-based bioresource enrichment analysis of differential gene sets was performed. The differential genes on the most relevant biological pathways were extracted, which is more conducive to the development of downstream experiments.

Using the “ClusterProfiler” R package to conduct KEGG functional enrichment analysis of co-expressed genes we found 14 KEGGs (P value < 0.05), and the enrichment table is presented as S6 File, while the KEGG bubble map is shown in Fig 2B [37].

### GSEA enrichment analysis

The high-risk and low-risk groups of samples were analyzed using GSEA enrichment (http://software.broadinstitute.org/gsea/index.jsp). First, we selected the gene set "c5.all.v6.2.symbols.gmt" and obtained the results of GO enrichment analysis. The enrichment table is given in S7 File and the enrichment graph is shown in Fig 2C. The results of the GSEA-GO analysis identified ten significantly altered pathways: cell cycle G1/S phase transition pathway, chromosome localization pathway, chromosome segregation pathway, condensed chromosome pathway, meiotic cell cycle process pathway, mitotic sister chromatid segregation pathway, nuclear chromosome segregation pathway, organelle fission pathway, sister chromatid cohesion pathway, and sister chromatid segregation pathway.

We selected the gene set "c2.cp.kegg.v6.2.symbols.gmt" to obtain a KEGG enrichment analysis. The enrichment table is listed in S8 File and the enrichment graph is presented as Fig 2D. The results of the GSEA-KEGG analysis revealed ten statistically significant pathways: cell cycle pathway, aminoacyl-tRNA-biosynthesis pathway, cysteine and methionine metabolism pathway, DNA replication pathway, oocyte meiosis pathway, pentose phosphate pathway, proteasome pathway, pyrimidine metabolism pathway, terpenoid backbone biosynthesis pathway, and vibrio cholera infection pathway.

### The results of cytological verification via Wound-healing and Transwell assays

**Fig 3:**
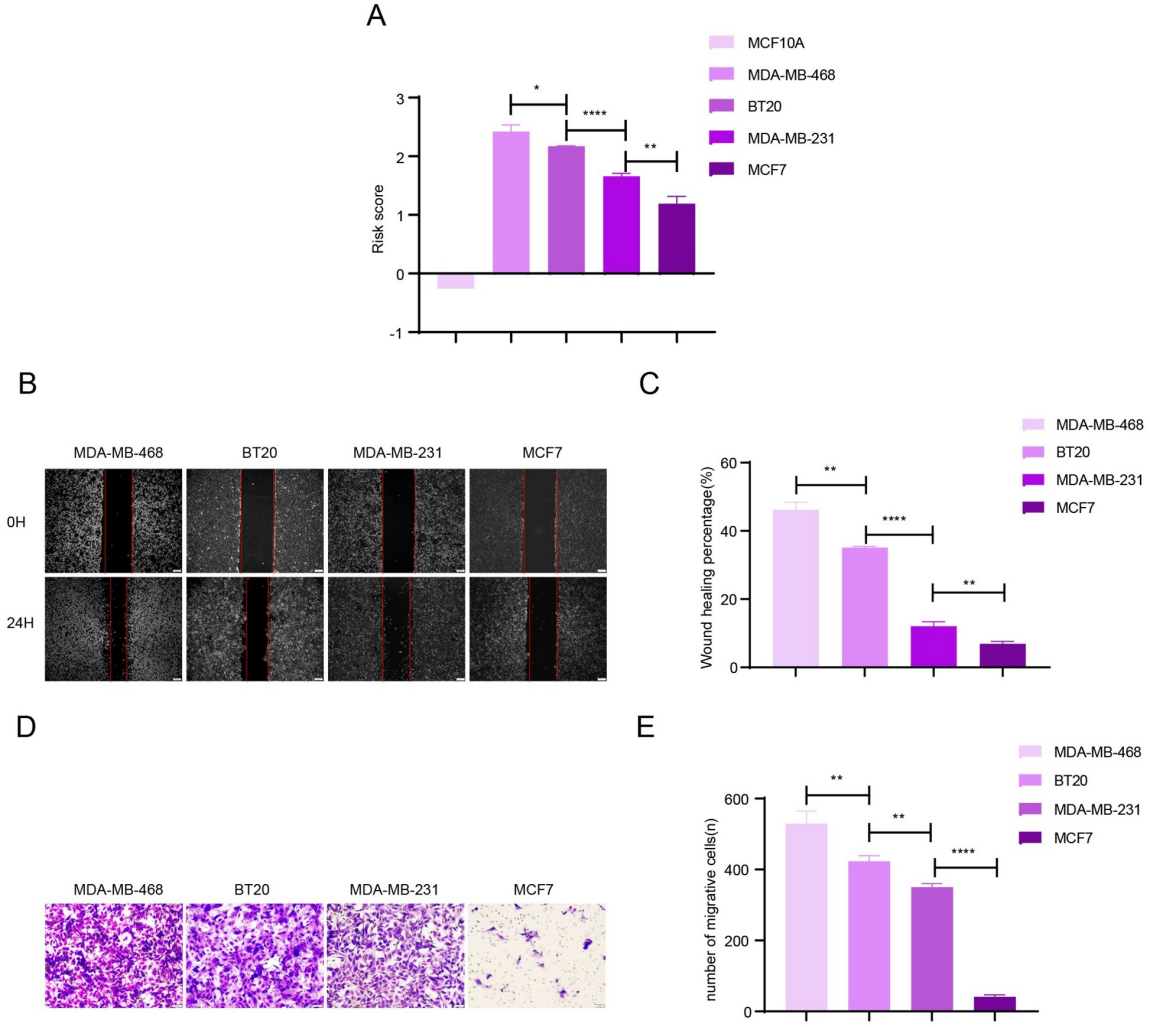
Validation of the risk signature with BCa cell lines. (A) The risk score calculated by the expression level of the 8 lncRNAs in 5 BCa cell lines. (B)-(E) The results of wound-healing and transwell assays using 4 BCa cell lines. The results showed that the cell lines with a higher risk score display stronger migration activity. Data are expressed as mean ± standard deviation (SD) (n = 3). *p < 0.1, **p < 0.01, ***p < 0.001, ****p < 0.0001.

Quantitative analysis of 8 lncRNAs of the 4 BCa cell lines and one normal breast cell line was conducted using the RT-PCR technique and the results are shown in S3A and S3B Fig. Using the formula to calculate risk score (Fig 3A), these 4 cell lines demonstrated different risk scores and then were used to conduct Wound-healing and Transwell assays, as the ANOVA p-value of the risk score is 6.196×10^−7^ among the four groups of breast cancer cells. Particularly, the risk score of the normal breast cell is lower than 0, indicating very low risk. In details, the p value comparing MDA-MB-468 to BT20 is 0.0193; the p value comparing BT20 to MDA-MB-231 is lower 0.0001; the p value comparing MDA-MB-231 to MCF7 is 0.0038. For Wound-healing assays, the p value comparing MDA-MB-468 with BT20 is 0.001; the p value comparing BT20 to MDA-MB-231 is lower 0.0001; the p value comparing MDA-MB-231 to MCF7 is 0.0038; and the ANOVA p-value is 1.042×10^−9^ among the four groups of breast cancer cells. For Transwell assays, the p value comparing MDA-MB-468 to BT20 is 0.0093; the p value comparing BT20 with MDA-MB-231 is 0.0022; the p value comparing MDA-MB-231 with MCF7 is lower 0.0001; and the ANOVA p-value is 1.128×10^−8^ among the four groups of breast cancer cells. The results presented in Fig 3B–3E showed that the cell lines with higher risk scores had greater migration ability than the cell lines with a lower risk score. This conclusion re-validates that the eight lncRNAs can be used as an assessment of the risk score and prognosis of BCa patients.

## Discussion

BCa has the highest morbidity and mortality among female malignancies worldwide [2]. A large proportion of BCa patients still suffer from local recurrence or distant metastasis even after comprehensive treatment [7]. Various genetic factors including lncRNA transcripts have been found to regulate the occurrence and development of breast cancer [34]. To date, lncRNAs have been used in the diagnosis, treatment, and as prognostic biomarkers of BCa, but most of these studies only focus on one or a small number of genes, and multi-gene analysis of prognosis is very rare [29, 38].

In the present study, we used a series of methods, including differential gene expression analysis, multivariate Cox regression analysis, correlation analysis, Chi-square tests, and subsistence analysis, to construct and validate an 8-lncRNA-based signature (MNX1-AS1, SIRLNT, AC092920.1, AC105219.1, AL355312.3, AC055854.1, LINC01117 and ACTA2-AS1) to predict BCa patient survival. MNX1-AS1, SIRLNT, AC092920.1, AC105219.1, AL355312.3 were classified as increased-risk genes, and AC055854.1, LINC01117, and ACTA2-AS1 were classified as protective genes. Using univariate Cox regression analysis, we found that only 3 of the lncRNAs (SIRLNT, AC092920.1, and AC055854.1) were independently correlated with OS. However, the risk score containing all 8 lncRNAs had a more significant relationship with OS of BCa patients (p<0.0001). In both univariate and multivariate Cox regression analysis, the values of HR were 1.069 and 1.077, respectively. In this study, the HR value of the survival analysis of biomarker is close to 1, which can be due to the good prognosis of breast cancer, which can be supported by the results of the survival analysis that the 5-year survival rate of breast cancer patients is more than 80%. However, this panel can greatly separate patients with high or low risk scores from KM survival analysis, and the P value of the survival analysis is lower than 0.0001, indicating the shows the statistically significant difference between the high and low risk score groups of breast cancer. Also, the results of breast cancer cell experiments show that breast cancer cells with higher risk score have stronger ability in cell proliferation and migration. In clinical practice, the treatment for patients with malignancy levels of cancer types is often radical and aggressive, while more conservative treatment options are often applied for patients with lower malignancy levels cancer types, including breast cancer. Therefore, this biomarker has the potential to predict the malignancy of breast cancer cells and suggest who might be benefited from radical treatment in advance, which is of significance to support the treatment of breast cancer patients. However, KM survival analysis based on the expression of single lncRNA indicates that any single lncRNA does not similar prognostic value as the model.

Therefore, a panel of lncRNAs was more sensitive and specific than each individual lncRNA and a risk score with a panel of 8 lncRNAs had a higher predictive value for the prognosis of BCa patients. Previous studies have shown that MNX1-AS1 is a risk factor for many cancers, such as ovarian cancer, laryngeal cancer, osteosarcoma, esophageal squamous cell carcinoma, gastric cancer, hepatocellular carcinoma, cervical cancer, and glioblastoma. MNX1-AS1 could promote osteosarcoma proliferation and invasion via inhibiting KISS1, migration and invasion of esophageal squamous cell carcinoma by upregulating IGF2, progression of cervical cancer through activating the MAPK pathway, and MNX1-AS1 overexpression promotes the invasion and metastasis of gastric cancer through repressing CDKN1A. Knockdown of LncRNA MNX1-AS1 inhibits proliferation, migration, and invasion of NSCLC cells and promotes apoptosis [18, 38–45]. ACTA2-AS1 is a protective factor that has been associated with diagnostic and prognostic value in ovarian cancer, BCa, and liver cancer patients [38, 46]. In this study, the signature we constructed using MNX1-AS1, SIRLNT, AC092920.1, AC105219.1, AL355312.3, AC055854.1, LINC01117 and ACTA2-AS1 was associated with BCa patients’ survival outcomes and migration of BCa cells.

The expression levels of the lncRNAs identified in this study could be used in clinical settings to calculate a patient’s risk score according to the formula developed in this study and this could help to determine whether the patient is at high risk or low risk and predict the patient’s possible survival time. According to our results, the five-year survival rate for the high-risk group is 79.1%, and for the low-risk group the five-year survival rate is 97.9%. This finding can provide guidance for the choice of clinical treatment plan and can also provide a reference for the patient’s own decisions, allowing for the formulation of a clinical treatment plan that is appropriate, improves the patient’s prognosis, and prolong survival time.

In our study, we found a set of 8 lncRNAs that can be used to predict the prognosis of BCa patients and we developed a formula that can be used as an independent prognostic biomarker for BCa patients. Furthermore, the obtained AUC value (AUC = 0.783) has a good accuracy for predicting the survival of BCa patients. Many patients have increasingly undergone genetic testing in clinical settings, and our results indicate that clinicians can make use of the results of such tests to assess the prognosis of individual BCa patients. Although the risk score developed in this study has a more accurate and specific predictive effect on the prognosis of BCa patients, there are still several limitations to note. First, the sample size of our study was limited. Since only those patients who had complete information were included in our study, there might be a selection bias in the primary cohort. Second, the biological functions of the 8 lncRNAs that we examined in BCa progression have not been revealed. Third, the exact roles and mechanisms of the identified lncRNAs in the development, progression, and treatment of BCa were not assessed with in vitro/vivo experiments and need to be further studied.

## Conclusion

In summary, we assessed 808 BCa patients from the TCGA data set to analyze the differential expression of lncRNA in normal and tumor tissues and found 8 lncRNA indicators that together can be used as an independent prognostic biomarker in BCa patients. This result was verified on four BCa cell lines using two in vitro cell migration assays. We also performed gene enrichment analysis on the possible pathways that are associated with the lncRNA identified in this study, but further validation is needed.

## Supporting information

S1 Fig KM curve based on the expression of single lncRNA. (TIF)

S2 Fig ROC analysis of single lncRNA. (TIF)

S3 Fig (A)(B) Expression of 8 lncRNAs in the indicated BCa cell lines by real-time PCR. Data are expressed as mean ± standard deviation (SD) (n = 3).
(TIF)

S1 Table The primer sequences used in this study. (DOCX)

S1 File The upregulated lncRNAs in breast tumor tissues compared to normal tonsil tissues. (XLSX)

S2 File The downregulated lncRNAs in breast tumor tissues compared to normal tonsil tissues. (XLSX)

S3 File The risk scores of all the breast cancer patients in this study. (XLSX)

S4 File The correlation analysis of the genes which have co-expression with the 8 lncRNAs. (XLSX)

S5 File GO enrichment analysis of the genes co-expressed with the 8 lncRNAs. (XLSX)

S6 File KEGG enrichment analysis of the genes co-expressed with the 8 lncRNAs. (XLSX)

S7 File GSEA_GO enrichment analysis of the genes co-expressed with the lncRNAs. (XLSX)

S8 File GSEA_KEGG enrichment analysis of the genes co-expressed with the lncRNAs. (XLSX)
