# Supplementary figures and images for: A panel of 8-lncRNA predicts prognosis of breast cancer patients and migration of breast cancer cells
Source: PLoS One. 2021 Jun 4;16(6):e0249174. doi: 10.1371/journal.pone.0249174 (PMC8177463; doi:10.1371/journal.pone.0249174)

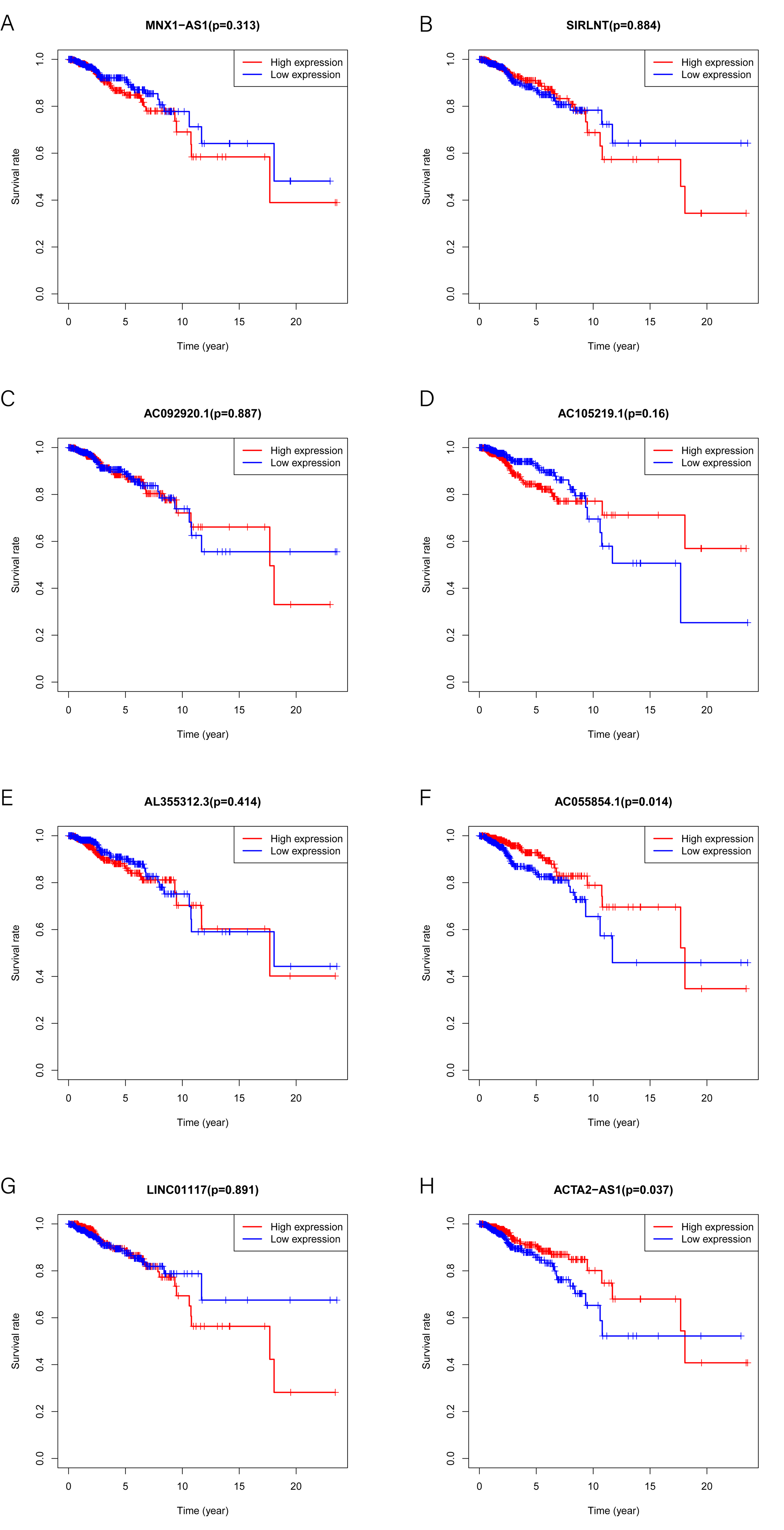

Supplement: S1 Fig — (TIF) [file pone.0249174.s001.tif]

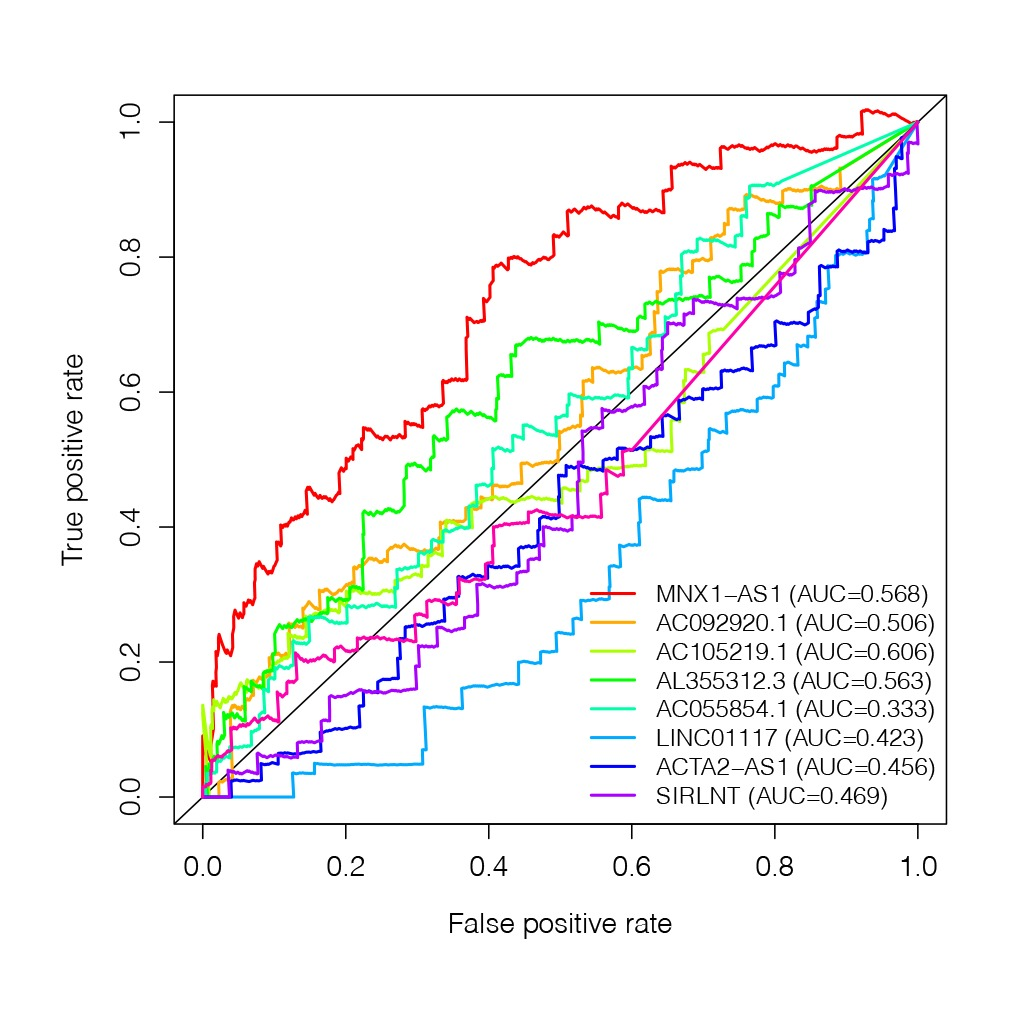

Supplement: S2 Fig — (TIF) [file pone.0249174.s002.tif]

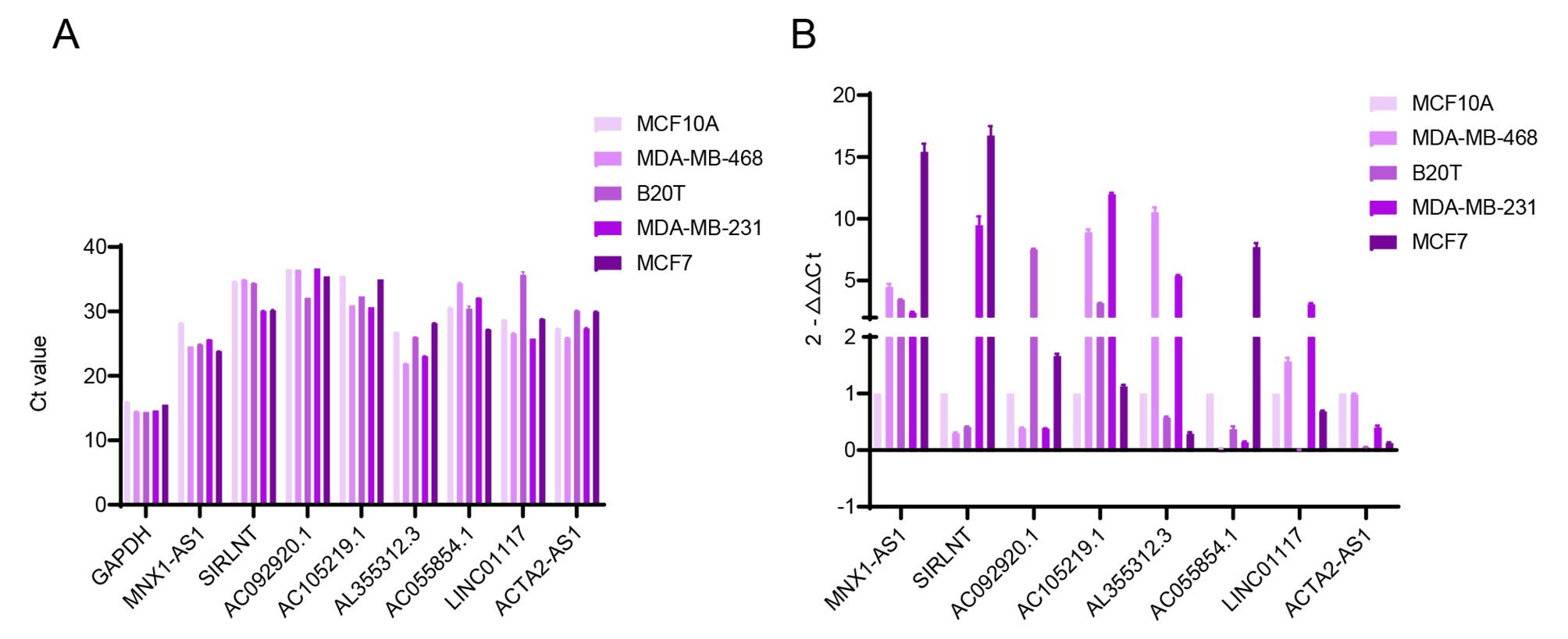

Supplement: S3 Fig — Data are expressed as mean ± standard deviation (SD) (n = 3). (TIF) [file pone.0249174.s003.tif]
