# Supplementary material for: A panel of 8-lncRNA predicts prognosis of breast cancer patients and migration of breast cancer cells
Source: PLoS One. 2021 Jun 4;16(6):e0249174. doi: 10.1371/journal.pone.0249174 (PMC8177463; doi:10.1371/journal.pone.0249174)
Supplement: S1 Table — (DOCX) [file pone.0249174.s004.docx]

**S1 Table. The Primer sequences included in this study.**

| **Gene** | **Primer sequences (5’ –3 ’)** |
| --- | --- |
| GAPDH-F | TGTTCGTCATGGGTGTGAAC |
| GAPDH-R | ATGGCATGGACTGTGGTCAT |
| MNX1-AS1-F | ACAACGCAGACAACATACAACT |
| MNX1-AS1-R | GCTTCTTCCCTGTGTGTTTCA |
| SIRLNT-F | CTTCTGGGTACACTTGGTCTTTC |
| SIRLNT-R | TTCATAATTGCCGTGCTCTTCA |
| AC092920.1-F | GGTGGTATCAGCAATTCAAGACT |
| AC092920.1-R | TGTTGTTACTCTGCCTCTTCCT |
| AC105219.1-F | GCTGCTGCTCTTAGTTGTCT |
| AC105219.1-R | CCTGCTAATCTTGGTGGACTT |
| AL355312.3-F | CAGTGACAACATGCGTTACCTT |
| AL355312.3-R | CACAGACGGGGTGGTTCTAG |
| AC055854.1-F | AGTTATTTAGCCTCCTGGATGTG |
| AC055854.1-R | CCTGCTCTTCTCATACGGATTG |
| LINC01117-F | GAAAGCAAGATGAAGTCACTGAGA |
| LINC01117-R | TGAAGGATATGTTGGGGTCTGT |
| ACTA2-AS1-F | GCCCATCAGGCAACTCGTAA |
| ACTA2-AS1-R | GCAGGTGAATCTTTGCCACC |
